# Supplementary material for: Splice-Junction-Based Mapping of Alternative Isoforms in the Human Proteome
Source: Cell Rep. Author manuscript; Available in PMC 2020 Jan 15. (PMC6961840; doi:10.1016/j.celrep.2019.11.026)
Supplement: 2 [file NIHMS1546469-supplement-2.zip › DF1/PXD006675/Aorta/Aorta_11_FHL1_GSSVVAYEGQSWHDYCFHCKKCSVCR.pdf]

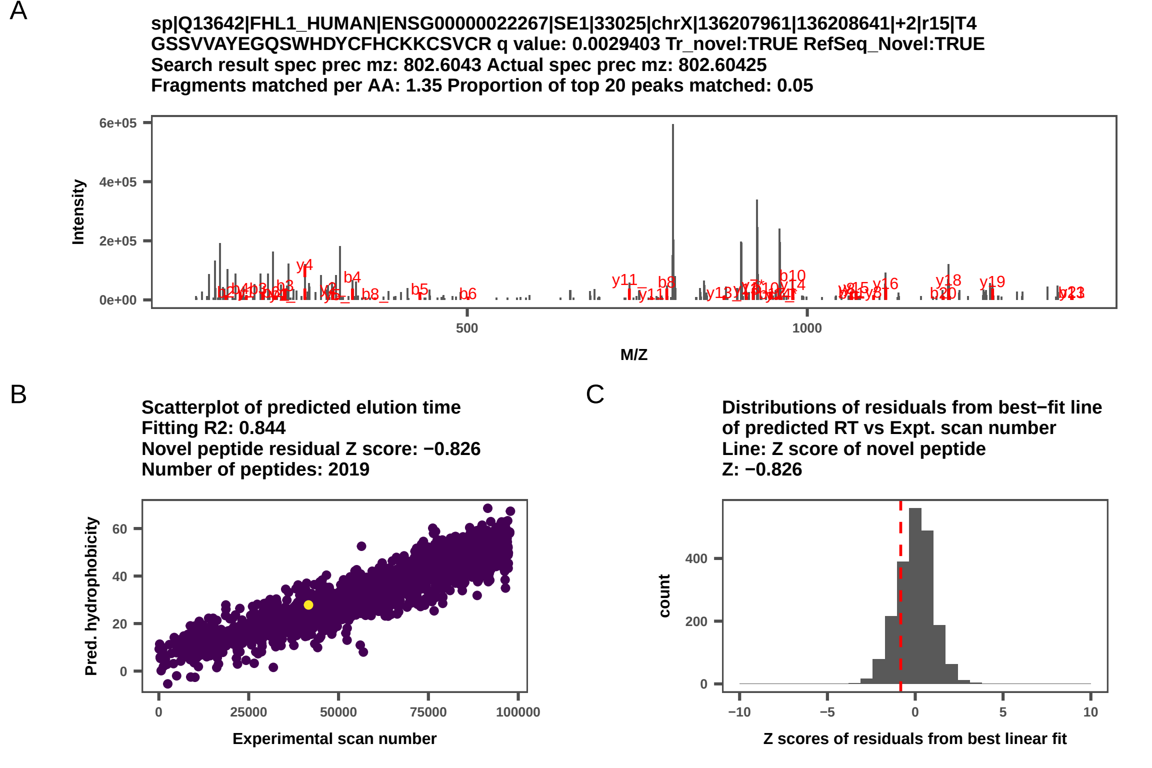
A
sp|Q13642|FHL1_HUMAN|ENSG00000022267|SE1|33025|chrX|136207961|136208641|+2|r15|T4
GSSVVAYEGQSWHDYCFHCKKCSVCR q value: 0.0029403 Tr_novel:TRUE RefSeq_Novel:TRUE
Search result spec prec mz: 802.6043 Actual spec prec mz: 802.60425
Fragments matched per AA: 1.35 Proportion of top 20 peaks matched: 0.05
Intensity
6e+05
4e+05
2e+05
0e+00
500
1000
M/Z
B
Scatterplot of predicted elution time
Fitting R2: 0.844
Novel peptide residual Z score: −0.826
Number of peptides: 2019
Pred. hydrophobicity
60
40
20
0
0
25000
50000
75000
100000
Experimental scan number
C
Distributions of residuals from best−fit line
of predicted RT vs Expt. scan number
Line: Z score of novel peptide
Z: −0.826
count
400
200
0
−10
−5
0
5
10
Z scores of residuals from best linear fit
